# Eudragit: A Novel Carrier for Controlled Drug Delivery in Supercritical Antisolvent Coprecipitation

**DOI:** 10.3390/polym12010234

**Published:** 2020-01-18

**Authors:** Paola Franco, Iolanda De Marco

**Affiliations:** Department of Industrial Engineering, University of Salerno, Via Giovanni Paolo II, 132, 84084 Fisciano, Italy; pfranco@unisa.it

**Keywords:** SAS coprecipitation, controlled drug delivery systems, microparticles, eudragit, supercritical carbon dioxide

## Abstract

In this work, the supercritical antisolvent (SAS) process was used to coprecipitate Eudragit L100-55 (EUD) with diclofenac (DICLO) and theophylline (THEOP), with the aim of obtaining composite microparticles with a prolonged drug release for oral delivery. Working at the optimized conditions in terms of pressure and overall concentration in the liquid solution (10.0 MPa and 50 mg/mL), microparticles of EUD/DICLO 20/1 and 10/1 *w*/*w* were produced with a mean size of 2.92 µm and 1.53 µm, respectively. For the system EUD/THEOP, well-defined spherical microspheres with a mean diameter ranging from 3.75 µm and 5.93 µm were produced at 12.0 MPa. The produced composite systems were characterized by various techniques, such as scanning electron microscopy, differential scanning calorimetry, X-ray microanalysis, FT-IR and UV–vis spectroscopy. Dissolution studies showed the potential of EUD to prolong the drug release, significantly, up to a few days.

## 1. Introduction

The inflammatory process is a defense response of body tissues against different harmful agents, irritants or pathogens. Inflammation can be acute when a rapid resolution is possible, or chronic when it is characterized by longer duration. Musculoskeletal disorders, such as rheumatoid arthritis and osteoarthritis, are chronic inflammations for which long-term therapies are required [1]. Non-Steroidal Anti-Inflammatory Drugs (NSAIDs) are generally used to reduce this kind of inflammatory conditions, as well as for their painkilling and antipyretic effects. Among the different drug delivery routes, oral administration is the preferred one because of its high patient compliance. Diclofenac (DICLO) is one of the most frequently prescribed NSAIDs; however, due to its short half-life, high dosage and frequent administration of diclofenac are necessary, resulting in many side effects, especially gastrointestinal ones. Another example of chronic inflammatory diseases is asthma, which affects the respiratory tract, due to an obstruction of the bronchi. Nowadays, the therapy goal is to achieve control of chronic bronchial asthma with fewer drug dosages. Therefore, drugs to be taken daily are recently prescribed in order to ensure long-term control of the symptoms and the maintenance of normal lung function. Theophylline (THEOP) is included in this category of drugs. However, high drug concentrations increase the risk of side effects (even toxicity effects), since the therapeutic range of this drug is very narrow [2]; to overcome the consequent limitations, prolonged-release formulations are preferred.

A valid solution to reduce the frequency of administration and unwanted effects is represented by controlled drug delivery systems. Generally speaking, the production of polymer/drug composite particles has a relevant importance in the pharmaceutical industry. By selecting a specific polymer as the carrier, it is possible to avoid the oxidation and/or the deactivation of the drug, to mask its organoleptic properties (color, taste, and odor) or modify its release kinetics. As a consequence, the bioavailability of a poorly water-soluble drug can be enhanced, or a prolonged drug release can be reached [3,4].

Spray-drying [5], freeze-drying [6], emulsification/solvent evaporation [7], centrifugal extrusion [8], jet-milling [9], and coacervation [10] are only some of the traditional techniques employed to produce polymer/drug coprecipitated powders. However, their application often involves the production of irregular particles with a wide particle size distribution (PSD), low encapsulation efficiencies, in addition to the possible degradation of the materials caused by mechanical or thermal stresses and/or organic solvents residues in the product. On the contrary, supercritical fluids (SCFs) based techniques seem to be a successful alternative to overcome these limits, especially when supercritical carbon dioxide (scCO_2_) is used, being non-toxic, non-polluting and cheap with moderate critical parameters (*T*_c_ = 31.1 °C, *P*_c_ = 7.38 MPa). Supercritical AntiSolvent (SAS) precipitation is one of the most used supercritical fluids based techniques, which has been successfully employed to obtain drug or polymer/drug nanoparticles, microparticles and expanded microparticles [11,12,13,14,15,16].

According to the SAS literature, in order to achieve a massive polymer/drug coprecipitation by the SAS process, microdroplets have to be formed, and after the subsequent removal of the solvent by scCO_2_, composite microspheres are produced.

However, the coprecipitation implies the formation of a quaternary system solvent + scCO_2_ + polymer + drug with a complex phase behavior, which can lead to the failure of the coprecipitation [16]. Up to now, a few polymers allow reaching a successful coprecipitation in the form of microspheres by the SAS process, namely, polyvinylpyrrolidone (PVP), poly (L-lactic acid) (PLLA), and recently, zein [11,12,17,18,19,20,21].

Commonly used for oral dosage forms, Eudragits are methacrylic acid copolymers, a registered trademark of Rohm Pharmaceuticals (Darmstadt, Germany). The various Eudragit polymers differ in the ratios of dimethylaminoethyl methacrylates, methacrylic acid and methacrylic esters. Eudragit E, RL, RS, and NE are polycations, because of the presence of dimethylamino groups or quaternary amino groups; whereas, Eudragit L and S are polyanions, due to the presence of carboxylate groups [22,23].

To our knowledge, very few literature studies were focused on the use of Eudragit polymers using the SAS process, although they are really interesting from a pharmaceutical point of view [13,14,24,25]. Indeed, they offer protection from moisture and masking of smells/flavors; moreover, they are versatile, since the choice of a specific Eudragit can be made based on the type of desired drug release (immediate, delayed or sustained). On the market, there are various kinds of Eudragits defined as pH-sensitive polymers, because they are soluble at different pH values [26,27]. Among them, Eudragit E100 and Eudragit EPO are soluble in the gastric juice up to pH 5.0, and therefore, are generally used for immediate release delivery systems [28]. Eudragit L100-55 (EUD) promotes a controlled drug release at pH higher than 5.5, starting from the first intestinal tract (duodenum), thus, protecting the drug against the acid gastric fluid [29,30]. Eudragit L100 differs from Eudragit L100-55 only for the presence of a methyl group instead of an ethyl group [31], which influences the slightly different dissolution pH threshold for the two polymers. Eudragit S100 is soluble at pH above 7.0; it can be used, in combination with other Eudragits, for colon-targeted oral drug delivery [32].

Montes et al. tried to coprecipitate Eudragit L100, which guarantees the drug release at pH values above 6, starting from the central section of the small intestine (jejunum), with ibuprofen [13], and naproxen [14] by SAS process. Eudragit L100/ibuprofen and Eudragit L100/naproxen particles with mean sizes in the range 0.08–0.51 µm and 0.08–0.31 µm, respectively were precipitated. Low loading efficiencies were achieved (0.94–7.88% for ibuprofen and 4.45–25.55% for naproxen), which can be attributed to the nanometric dimension of the powders; indeed, it is presumable that the particles at nanodimensions tend to precipitate separately, considering that it is difficult to obtain heterogeneous nucleation. Similar results were obtained by the same authors in another study [33], coprecipitating Eudragit L100 and ellagic acid at different polymer/drug ratios. From the shown FESEM images, it is possible to observe that the precipitation of crystals occurred using Eudragit L100/ellagic acid ratios equal to 1/1 and 2/1, whereas, coalescent sub-microparticles precipitated using a polymer/drug ratio of 4/1. Garay et al. processed Eudragit EPO using the gas antisolvent process [25]; using EtOH/H_2_O mixtures as the solvent, they obtained micrometric particles with a wide particle size distribution. Duarte et al. coprecipitated acetazolamide with Eudragit L100, Eudragit S100, and their mixtures [24]; the SEM images reported in the paper show the co-presence of crystals and microparticles; this suggests that the polymer and the drug precipitated separately and that an effective coprecipitation did not occur.

Up to now, it would appear that SAS coprecipitation using Eudragit as the polymeric carrier is not successful. Therefore, with the aim of obtaining prolonged drug delivery devices for oral administration, Eudragit L100-55 was selected as the polymeric carrier for SAS coprecipitation. Firstly, the effect of some process conditions on the micronization of Eudragit L100-55 (without the drug) was studied to obtain spherical microparticles. Then, several SAS coprecipitation experiments were performed using an NSAID (diclofenac) and a bronchodilator drug (theophylline) as model compounds.

## 2. Materials, Methods and Procedures

### 2.1. Materials

Eudragit L100-55 (EUD) was generously donated by Degussa (Darmstadt, Germany). Diclofenac sodium salt (DICLO, average molecular weight 318.13 g/mol), anhydrous theophylline (THEOP, average molecular weight 180.16 g/mol) and Dimethylsulfoxide (DMSO, purity 99.5%) were purchased by Sigma-Aldrich (Milan, Italy). CO_2_ (purity 99%) was supplied from Morlando Group s.r.l. (Sant’Antimo - NA, Italy). All materials were used as received. The solubility in DMSO at room temperature is approximately 55 mg/mL for EUD, 150 mg/mL for DICLO and 25 mg/mL for THEOP.

### 2.2. SAS Apparatus and Procedure

**Figure 1:**
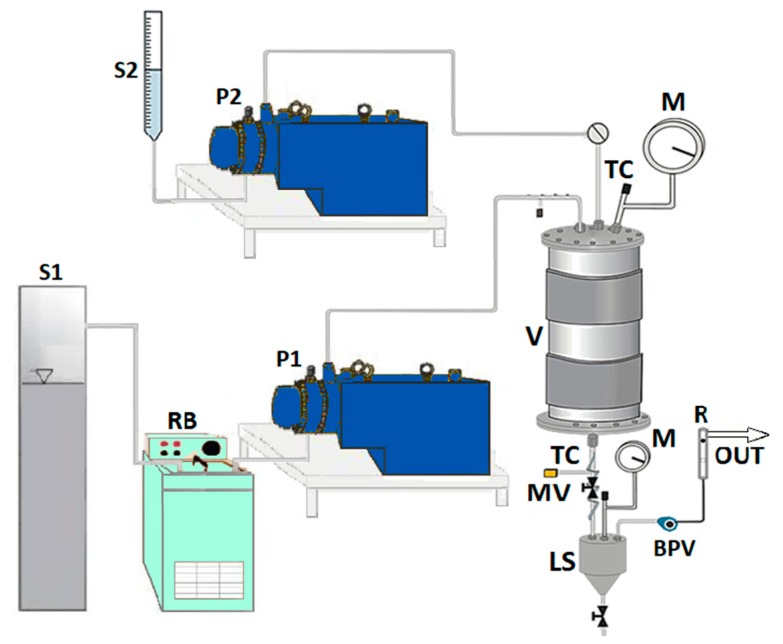
A sketch of the supercritical antisolvent (SAS) laboratory plant. S1, tank for the CO_2_; S2, organic solution; RB, refrigerating bath; P1, P2, pumps; V, vessel; M, manometer; TC, thermocouple; MV, micrometric valve; LS, liquid separator; BPV, back-pressure valve; R, rotameter.

The homemade laboratory plant used to perform SAS experiments is sketched in Figure 1. 

The precipitation vessel (V) is a cylindrical chamber with an internal volume of 500 cm^3^. The CO_2_ is stored in a tank (S1) and feed to the vessel through a high-pressure pump (P1). The liquid solution contained in a burette (S2) is co-currently injected into the vessel by another high-pressure pump (P2), passing through a 100 µm internal diameter stainless steel nozzle. The temperature control (TC) is assured by a proportional integral derivative (PID) controller connected with electrically thin bands and the pressure in the vessel is measured using a test gauge manometer (M) and regulated by a micrometric valve (MV). At the bottom of the precipitator, the precipitated powder is collected on a stainless steel filter (pores size of 0.1 μm), which also allows the passage of CO_2_—solvent solution. Downstream the precipitation chamber, the liquid solvent is recovered in a second collection vessel (liquid separator, LS) at a lower pressure (1.8–2.0 MPa) regulated by a back-pressure valve (BPV). The flow rate of delivered CO_2_ is measured at the exit of the second vessel by a rotameter ®.

A SAS test starts pumping CO_2_ at a constant flow rate in the precipitation vessel until the desired pressure is reached; then, the antisolvent steady flow is established. After that, pure solvent (DMSO) is injected through the nozzle in co-current mode with CO_2_, since the steady-state solvent/antisolvent composition is reached. Then, the solvent flow rate is stopped, and the liquid solution (DMSO + solutes) is injected into the vessel at the same flow rate as the pure DMSO with consequent precipitation of the solute/solutes. At the end of the solution injection, the liquid pump is stopped, and only the scCO_2_ is sent to the precipitation chamber to eliminate the solvent residues. The CO_2_ flow is stopped when the washing step is completed, and the precipitator is depressurized up to the atmospheric pressure. Finally, the precipitated powder can be collected and characterized.

All SAS experiments were performed using an operating temperature of 40 °C, DMSO as the liquid solvent, a CO_2_ flow rate and a solution flow rate equal to 30 g/min and 1 mL/min, respectively. At the selected temperature, the flow rates were chosen to obtain molar fractions on the right of the mixture critical point (MCP) of the binary system solvent/antisolvent; i.e., at CO_2_ molar fractions approximately equal to 0.98 that ensures the supercritical mixture conditions [34,35]. In the case of EUD micronization, the effect of the operating pressure (P) and of the overall concentration in the liquid solution (C_tot_) on particles’ morphology, particle mean diameter (m.d.) and particle size distribution (PSD) was studied; in the case of EUD/DICLO and EUD/THEOP coprecipitation, the effect of the polymer/drug ratio, P and C_tot_ on morphology, m.d., and PSD was investigated.

### 2.3. Characterization Methods

The morphology of the samples was evaluated by a Field Emission Scanning Electron Microscope (FESEM, mod. LEO 1525, Carl Zeiss SMT AG, Oberkochen, Germany). The powder was dispersed on a carbon tab previously stuck to an aluminum stub (Agar Scientific, Stansted, United Kingdom) and coated with gold-palladium (layer thickness 250 Å) using a sputter coater (mod. 108 A, Agar Scientific, Stansted, United Kingdom). Mean dimensions and standard deviations of particles were measured from FESEM photomicrographs by using an image analysis software (SigmaScan Pro, Jandel Scientific, Bangalore, India), considering about 1000 particles for each sample. Particle size distributions (PSDs) were determined by Microcal Origin Software (release 8.0, Microcal Software, Inc., Northampton, MA, USA).

Fourier transform infrared (FT-IR) analyses were performed by M2000 FT-IR (MIDAC Co, Costa Mesa, CA, USA). The scan wavenumber range was 4000–450 cm^−1^, at a resolution of 0.5 cm^−1^ and 16 scan signals were averaged to reduce the noise. Approximately 1 mg of the powder samples was well-mixed with 100 mg of potassium bromide (KBr), which was used as an infrared transparent matrix. The discs to be analyzed were prepared by compressing the powders in a hydraulic press.

Calorimetric analyses were obtained by means of a Differential Scanning Calorimeter (DSC, mod. TC11, Mettler-Toledo, Inc., Columbus, OH, USA) using the Mettler STARe system. The accurately weighed powders (5 ± 0.5 mg) were heated from 25 °C up to 300 °C, using a heating rate of 5 °C/min and a nitrogen gas flow equal to 50 mL/min. Each analysis was performed twice.

An X-ray diffractometer (Bruker D8-Advance, Bruker AXS GmbH, Karlsruhe, Germany) with a Cu sealed tube source was used to record the X-ray diffractograms (XRD) of samples. The measuring conditions were: Ni-filtered Cu*K_α_* radiation, λ = 1.54 Å, 2θ angle ranging from 10° to 60° with a scan rate of 0.5 s/step and a step size of 0.08°.

In vitro drug dissolution was monitored using a UV/vis spectrophotometer (model Cary 50, Varian, Palo Alto, CA, USA) at a wavelength of 276 nm and 271 nm for DICLO and THEOP, respectively. An equivalent amount of drug equal to 5 mg was considered to compare the dissolution rate of unprocessed drug and that of coprecipitated powders. The samples were suspended in 3 mL of phosphate-buffered saline solution (PBS) at pH 7.4 and placed into a 12,000 Da molecular weight cutoff dialysis sack, which was incubated in 300 mL of PBS, continuously stirred at 200 rpm and heated at 37 ± 0.1 °C. The value of the absorbance was measured by the instrument every minute from time zero up to 5 h, then every hour until the maximum value was reached. Each analysis was performed in triplicate: Considering that the difference between the triplicates was less than 1%, the mean release profiles were reported in this paper.

## 3. Results and Discussion

A summary of several experiments performed at different process conditions is reported in Table 1, indicating the obtained morphology (M), the mean diameter (m.d.) and the standard deviation (s.d.) on a volumetric basis. In particular, some results reported in Table 1 are related to the micronization of EUD alone, followed by coprecipitation studies on the systems EUD/DICLO and EUD/THEOP.

### 3.1. Micronization of Eudragit L100-55

The first set of experiments (runs #1–4 in Table 1) was performed by micronizing EUD alone, at different P and C_tot_, in order to optimize the process conditions for the attainment of polymer microparticles. 

#### Effect of the Operating Pressure

The effect of the operating pressure was investigated setting a polymer concentration in DMSO equal to 20 mg/mL and gradually increasing the pressure from 9 to 12 Mpa (runs #1–3 in Table 1).

**Figure 2:**
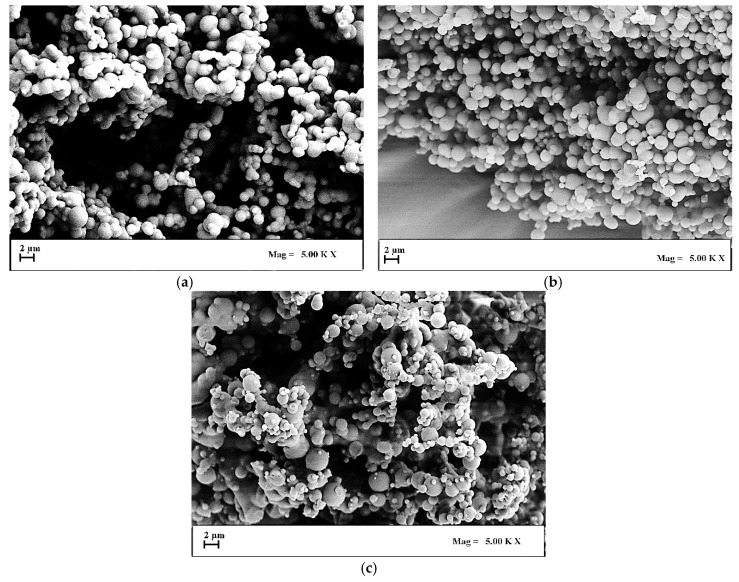
FESEM images of Eudragit particles precipitated from DMSO at 40 °C and 20 mg/mL. Effect of the operating pressure. (**a**) 9 MPa; (**b**) 10 MPa; (**c**) 12 MPa.3.1.2 Effect of polymer concentration in DMSO.

FESEM analyses revealed that well-separated spherical microparticles were obtained working at 9 and 10 Mpa (Figure 2a,b, respectively), whereas, coalescing and more irregular in shape and dimensions microparticles were formed at 12 Mpa (Figure 2c). Moreover, a slight increase of the particle dimensions was observed by decreasing the pressure, as shown in the last column of Table 1. Considering that it has been previously demonstrated that SAS coprecipitation is favored at pressures in correspondence of which well-defined micrometric particles are produced, for the subsequent experimentation the pressure of 12 Mpa was not considered [16].

**Figure 3:**
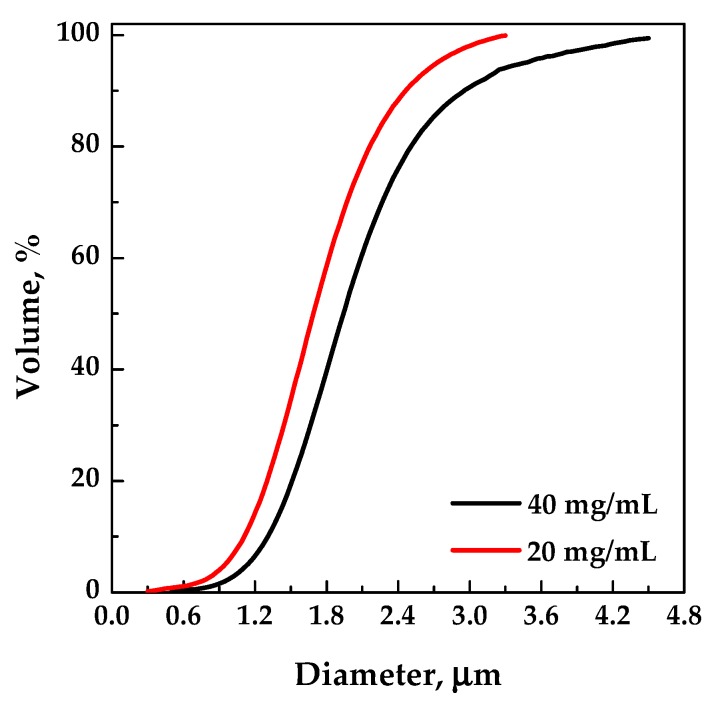
Volumetric cumulative particle size distributions (PSDs) of EUD precipitated from DMSO at 40 °C and 10 MPa; effect of the polymer concentration in DMSO.

Then, fixing the pressure at 10 MPa, the polymer concentration in DMSO was increased at 40 mg/mL (run #4 in Table 1). Spherical microparticles were produced; the comparison of the volumetric cumulative PSDs in Figure 3 (related to runs #2 and #4) demonstrated that the mean particle size increased and the particle size distribution widened by increasing the EUD concentration in DMSO. Indeed, the higher the concentration of the polymer in the liquid solution, the more the phenomenon of growth prevails over that of nucleation.

### 3.2. Coprecipitation Using Eudragit as the Carrier

Once verified the attainment of EUD microparticles using the SAS process, the polymer coprecipitation was attempted using diclofenac and theophylline as model drugs.

**Figure 4:**
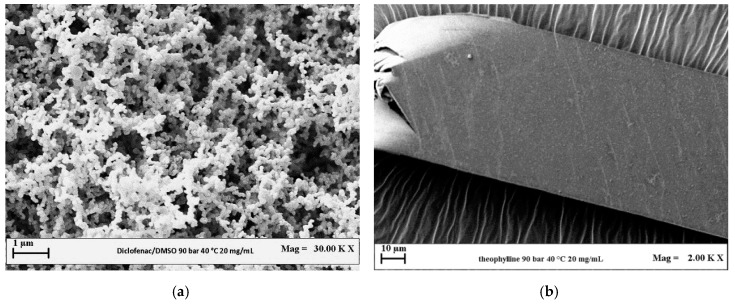
FESEM images of the drugs precipitated from DMSO at 9 MPa, 40 °C and 20 mg/mL. (**a**) DICLO; (**b**) THEOP.

Preliminary experiments were performed by processing each drug separately at 40 °C, 9 MPa using a solute concentration in DMSO equal to 20 mg/mL (runs #5 and #11 in Table 1). From FESEM analyses, it is possible to note that SAS processed diclofenac (Figure 4a) precipitated in the form of nanoparticles (mean diameter of about 0.13 µm). On the contrary, after processing theophylline alone, no powder was found in the precipitation chamber, because it was probably extracted by the mixture formed by the organic solvent and supercritical CO_2_. Long crystals (Figure 4b) were observed by analyzing the very few granules that were recovered.

In order to successfully coprecipitate the two model drugs with EUD, the effect of pressure, of polymer + active principle concentration in DMSO and of polymer/drug ratio *w*/*w* on the morphology and on the mean size of the produced composite particles was investigated.

#### 3.2.1. Effect of the Operating Pressure on Coprecipitated Particles

**Figure 5:**
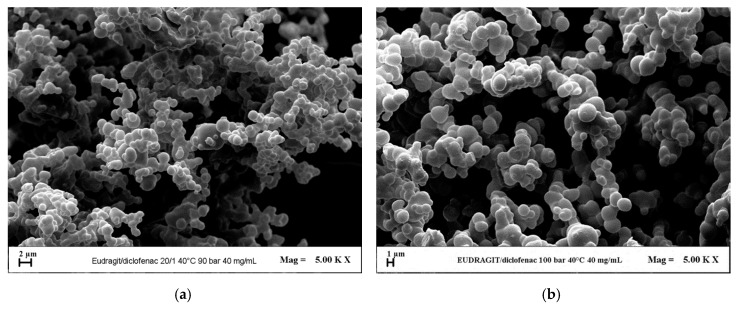
FESEM images of EUD/DICLO 20/1 particles precipitated from DMSO at 40 °C and 40 mg/mL. Effect of the operating pressure. (**a**) 9 MPa (run #6); (**b**) 10 MPa (run #7).

Firstly, the effect of the pressure was investigated for the system EUD/DICLO by selecting 9 and 10 MPa as pressures, according to the previous results obtained by studying EUD micronization. Moreover, an overall concentration of solutes in DMSO of 40 mg/mL and a polymer/drug ratio equal to 20/1 *w*/*w* were fixed. A less degree of coalescence of the particles was observed operating at 10 MPa (run #7 in Table 1) with respect to 9 MPa (run #6 in Table 1), as it is possible to observe from the FESEM images reported in Figure 5.

**Figure 6:**
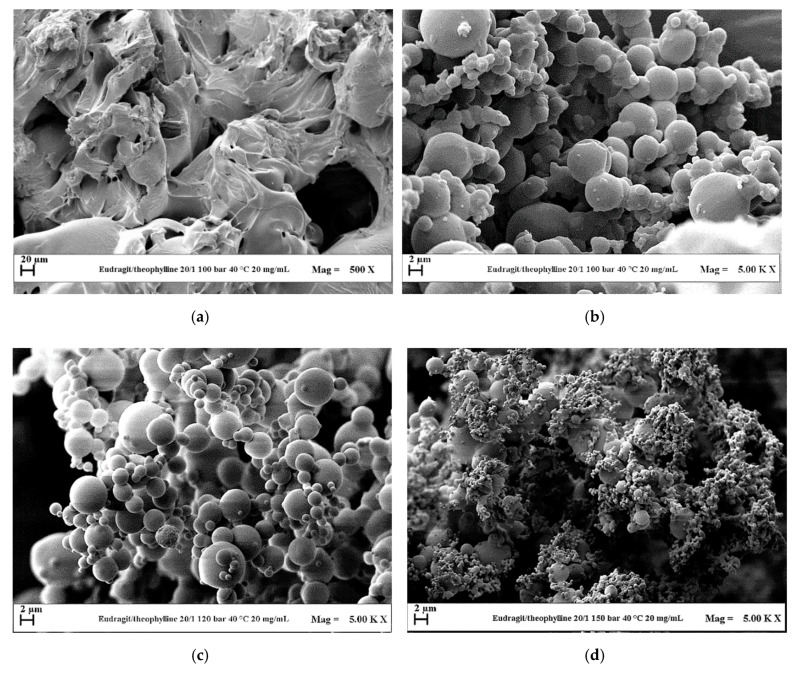
FESEM images of EUD/THEOP 20/1 powders precipitated from DMSO at 40 °C and 20 mg/mL. Effect of the operating pressure. (**a**) 10 MPa (run #13) filter; (**b**) 10 MPa (run #13) precipitating chamber; (**c**) 12 MPa (run #14); (**d**) 15 MPa (run #15).

Considering that the pressure of 10 MPa gave the best results in terms of sphericity and mean diameter of the powders in the case of EUD micronization and for its coprecipitation with DICLO, the preliminary tests with the system EUD/THEOP 20/1 *w*/*w* at 40 mg/mL (run #12) and 20 mg/mL (run #13) were performed in correspondence of this pressure. Using this system, crystals and crystals precipitated with coalescing microparticles (as shown in Figure 6a,b for run #13) were obtained at 40 mg/mL and 20 mg/mL, respectively. This outcome may be explained, taking into account the thermodynamic aspects. Considering the temperature of 40 °C, the mixture critical point (MCP) of the binary system DMSO/CO_2_ is located at 8.61 MPa [34]; it means that the operating point at 10 MPa is above the MCP. However, the presence of the solutes can modify the high-pressure vapor-liquid equilibria (VLEs) of the system, and the MCP of the quaternary system EUD/THEOP/DMSO/scCO_2_ could shift towards higher pressures with respect to the one of the binary system formed by the solvent and the antisolvent. This shift of the MCP towards higher pressures is generally more evident by increasing the concentration of the solutes in the liquid solution [36,37]. In this specific case, the presence of THEOP probably modified the high-pressure VLEs; thus, the operating point at 10 MPa could be below the MCP and lie in the biphasic region, from which a split of the precipitated solute in two morphologies may occur; i.e., crystals and microparticles as in case of run #13.

**Figure 7:**
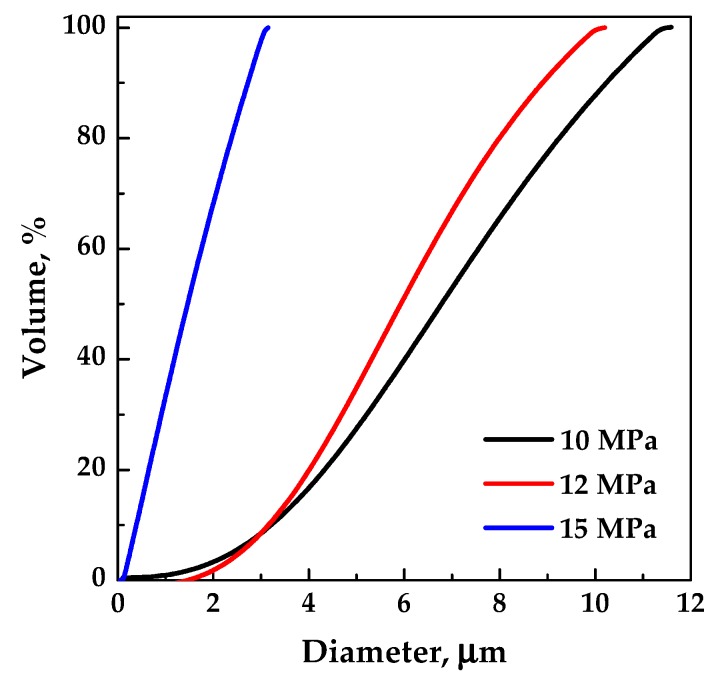
Volumetric cumulative PSDs of EUD/THEOP 20/1 particles precipitated from DMSO at 40 °C and 20 mg/mL; effect of the operating pressure.

In the following set of experiments, with the aim of shifting the operating point above the MCP, the total concentration in DMSO was fixed at 20 mg/mL, and the effect of pressure in the range 10–15 MPa (runs #13–15) was evaluated. Well-defined spherical microparticles (Figure 6c) were obtained in correspondence of a pressure of 12 MPa, whereas, slightly coalescing microparticles precipitated at 15 MPa (Figure 6d). Moreover, it was observed that the particles mean diameter decreased by increasing the pressure, as shown by the comparison of the volumetric cumulative PSDs reported in Figure 7.

#### 3.2.2. Effect of Total Concentration on Coprecipitated Particles

**Figure 8:**
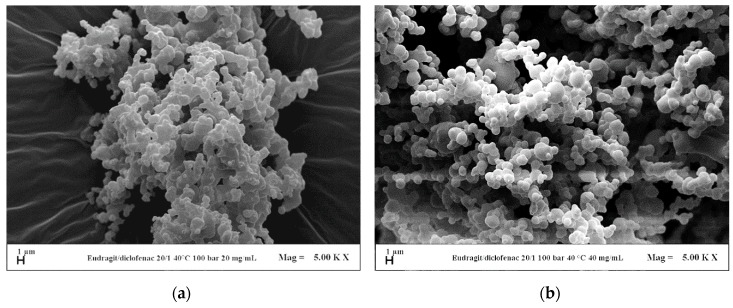
FESEM images of EUD/DICLO 20/1 particles precipitated from DMSO at 40 °C, 10 MPa. (**a**) 20 mg/mL (run #8); (**b**) 50 mg/mL (run #9).

Since the pressure of 10 MPa allows the attainment of defined EUD/DICLO particles, the influence of the overall concentration in DMSO at a polymer/drug ratio of 20/1 *w*/*w* was evaluated in correspondence of that pressure. Coalescing particles (Figure 8a) precipitated at 20 mg/mL (run #8), whereas, less coalescing particles were obtained by increasing the total concentration up to 50 mg/mL (run #9), as shown in Figure 8b.

For the system EUD/THEOP 20/1 *w*/*w*, the effect of the overall concentration in DMSO was evaluated at 12 MPa in the range 20 mg/mL (run #14)–40 mg/mL (run #16). Well-defined spherical microparticles were obtained at 40 mg/mL, as well as at 20 mg/mL; no significant changes in terms of particle diameters occurred by increasing the concentration.

#### 3.2.3. Effect of Polymer/Drug Ratio on Coprecipitated Particles

For the system EUD/DICLO, the pressure was fixed at 10 MPa and the total concentration at 50 mg/mL; in correspondence of these conditions, the polymer/drug ratio was decreased from 20/1 to 10/1 *w*/*w*. Slightly irregular and coalescing microparticles were produced working at 10/1 *w*/*w*, and a decrease of the mean diameter was observed by decreasing the polymer/drug ratio.

**Figure 9:**
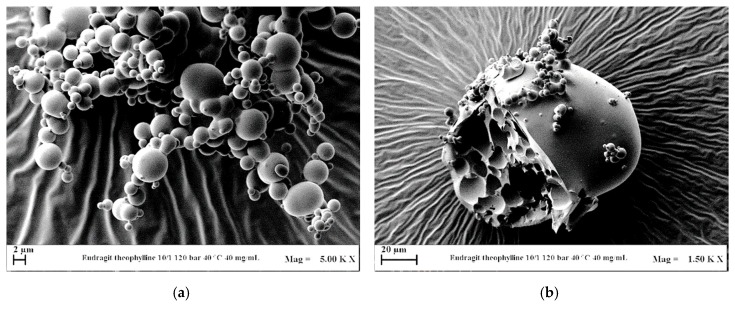
FESEM images of EUD/THEOP 10/1 *w*/*w* precipitated from DMSO at 40 °C, 12 MPa and 40 mg/mL (run #17). (**a**) Microparticles and (**b**) expanded microparticles.

**Figure 10:**
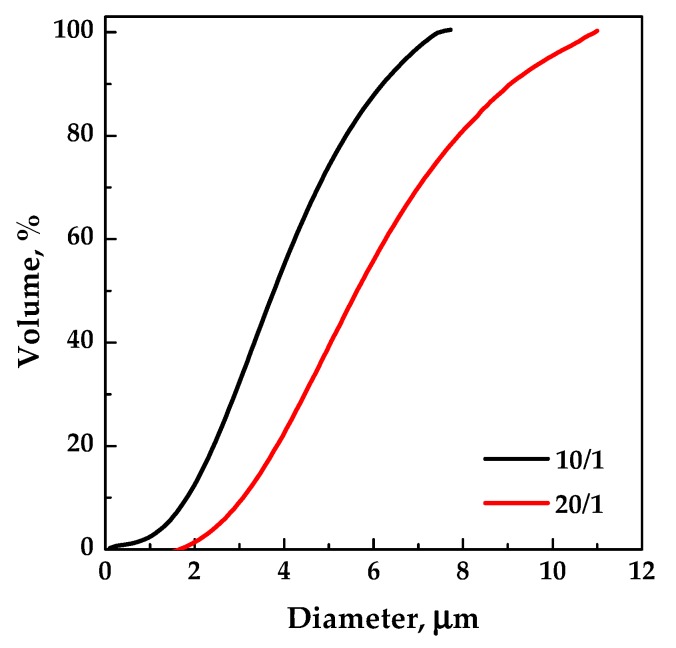
Volumetric cumulative PSDs of EUD/THEOP particles precipitated from DMSO at 40 °C, 12 MPa and 40 mg/mL; effect of the polymer/drug ratio.

EUD/THEOP ratio *w*/*w* was instead decreased from 20/1 to 10/1 *w*/*w*, fixing the pressure at 12 MPa and the total concentration at 40 mg/mL. Well-defined microparticles (Figure 9a) were obtained even at 10/1 *w*/*w* (run #17 in Table 1), with a lower average diameter than the particles produced at 20/1, as shown from the comparison of the volumetric cumulative PSDs reported in Figure 10. The presence of few larger particles (Figure 9b) with an internal structure characterized by holes was also noted working at 10/1 *w*/*w*. This outcome can be ascribed to the stronger influence of theophylline on the VLEs, since a higher concentration in the liquid solution can cause the MCP shift at higher pressures.

### 3.3. Characterization of Samples

**Figure 11:**
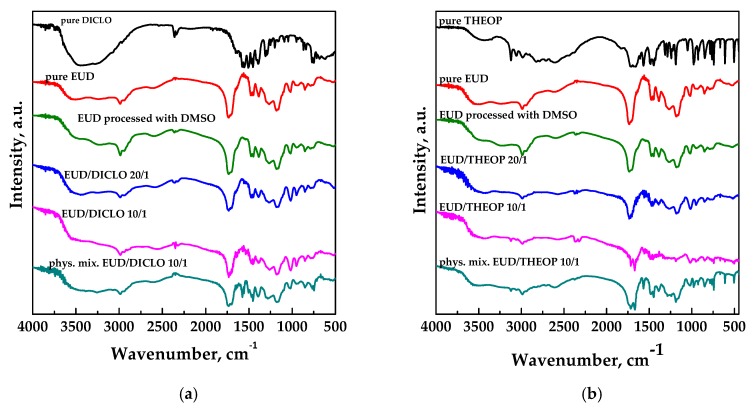
FT-IR spectra for unprocessed and SAS processed Eudragit L100-55, unprocessed drugs, physical mixture polymer/drug and SAS processed Eudragit/drug powders. (**a**) DICLO; (**b**) THEOP.

Fourier transform infrared (FT-IR) analyses were performed in order to identify the presence of the polymer and the drug in the composite powders and possible interactions between the two compounds. FT-IR spectra of unprocessed drug, unprocessed and processed EUD, physical mixture EUD/drug 10/1 *w*/*w* and SAS processed EUD/drug 20/1, and 10/1 are reported in Figure 11a,b for DICLO and THEOP, respectively. 

FT-IR spectra of pure and unprocessed EUD is characterized by absorption bands at around 1157 cm^−1^, 1184 cm^−1^ e 1261 cm^−1^, corresponding to the ester vibrations, peaks at about 1701 cm^−1^ and at 1736 cm^−1^ attributed to the C = O stretching of the carboxylic acid and the vibrations of the esterified carboxyl groups, respectively; moreover, the presence of peaks at 1387 cm^−1^, 1479 cm^−1^ e 2979 cm^−1^ corresponded to CHX vibrations [29]. No changes were observed in the polymer at the level of the functional group after SAS processing. FT-IR spectra of the physical mixture and SAS processed EUD/DICLO powders clearly showed the characteristic bands of the polymer, since it is present in more quantity, and few peaks related to DICLO as the C–Cl stretching at about 720 cm^−1^ [38]. In addition to EUD characteristic peaks, FT-IR spectra of physical mixture and SAS processed EUD/THEOP powders exhibited several absorption bands attributed to the theophylline, such as at 3120 cm^−1^ assigned to the N–H stretching, at 3060 and 2989 cm^−1^ related to the C–H stretching, at about 1718 and 1667 cm^−1^ assigned to the carbonyl stretching and at 1307 cm^−1^ assigned to the C–O stretching [39,40,41]. 

**Figure 12:**
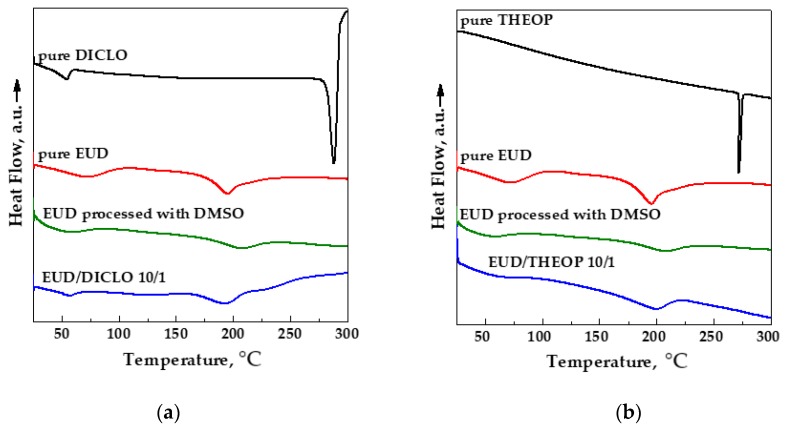
DSC thermograms of unprocessed and SAS processed EUD, unprocessed drugs, and SAS processed EUD/drug coprecipitated powders. (**a**) DICLO; (**b**) THEOP.

DSC thermograms of the unprocessed drug, unprocessed and processed EUD and SAS processed EUD/drug 20/1 and 10/1 *w*/*w* are reported in Figure 12a,b for DICLO and THEOP, respectively. 

The DSC curve of pure DICLO showed two endothermic peaks, the first one related to the dehydration and the second one at about 288 °C corresponding to the melting point [42]. The DSC thermogram of pure THEOP showed an endothermic peak at around 272 °C, which is its melting point [43]. The thermogram of unprocessed EUD had two endothermic peaks, the first one, ascribable to the loss of water and the other one at about 200 °C related to the melting of the crystalline portion of the polymer [44]. Moreover, in agreement with the literature [45], unprocessed EUD is characterized by a glass transition temperature (*T_g_*) at around 119 °C, slightly shifted at a lower temperature (around 112 °C) for SAS processed EUD, because of the scCO_2_ plasticizing effect in the presence of semicrystalline polymers [46,47]. The thermal behavior of SAS coprecipitated powders is similar to the polymer one. The absence of the melting point of the drug in the thermograms of the coprecipitated powders can be ascribed to the amorphization. Moreover, because of the particle size reduction, a slightly higher degree of amorphism is detected both in SAS processed polymer and SAS coprecipitated powders with respect to unprocessed EUD, as observable from the less pronounced melting peak linked to the crystalline portion of the polymer.

**Figure 13:**
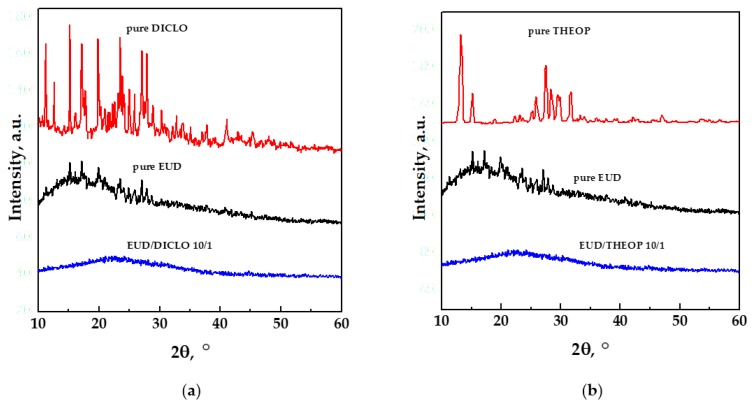
XRD patterns of unprocessed EUD, unprocessed drugs, and SAS processed EUD/drug coprecipitated powders. (**a**) DICLO; (**b**) THEOP.

XRD patterns of the unprocessed drug, unprocessed EUD and SAS processed EUD/drug 10/1 *w*/*w* are reported in Figure 13a,b for DICLO and THEOP, respectively. 

XRD analyses confirmed the outcomes deduced by DSC thermograms; i.e., the pure drugs were in a crystalline state, the pure EUD showed a semi-crystalline pattern, whereas, the coprecipitated powders were characterized by an amorphous behavior.

**Figure 14:**
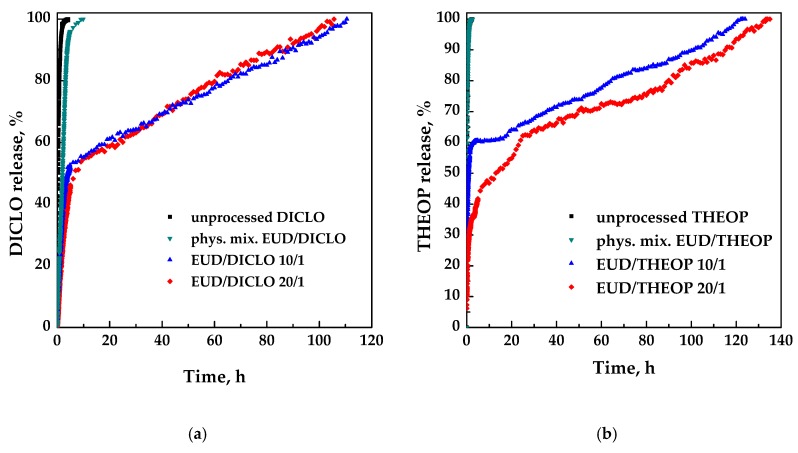
Dissolution profiles in PBS at 37 °C and pH 7.4. (**a**) DICLO; (**b**) THEOP.

Dissolution tests were performed using UV-vis spectroscopy to compare the dissolution rate of each unprocessed drug with the drug coprecipitated with EUD. The dissolution profiles in PBS of the unprocessed drug, physical mixture EUD/drug 10/1 *w*/*w* and SAS processed EUD/drug 20/1, and 10/1 are reported in Figure 14a,b for DICLO and THEOP, respectively. 

Pure DICLO and DICLO present in the physical mixture were completely released in about 4 h and 10 h, respectively. The delayed effect of the drug release, due to the coprecipitation with the polymer is evident since the complete dissolution is achieved for both SAS coprecipitated powders 20/1 and 10/1 in about 100 h with a similar burst effect (about 45%) ascribable to the drug portion located near/on the surface of the particles. In summary, the dissolution of DICLO coprecipitated with EUD was about 28 times slower with respect to the unprocessed NSAID. 

As for pure theophylline and the corresponding physical mixture, the time taken for the complete release is practically the same (about 2.3 e 2.6 h, respectively). Differently, SAS coprecipitated EUD/theophylline 10/1, and 20/1 *w*/*w* powders require for the complete drug dissolution about 118 h and 130 h, respectively; therefore, also, in this case, the release was considerably prolonged using EUD, up to about 57 times. Moreover, by increasing the polymer/drug ratio from 10/1 to 20/1 *w*/*w*, the drug release burst decreased from 60% to 20%. The difference in the release of EUD/THEOP 10/1 and 20/1 *w*/*w* can be explained, according to the literature [48], to the size of the microparticles. Indeed, increasing the powders’ diameter, the portion of the drug finely dispersed in the polymer microparticle increased, and correspondingly, the portion of the drug disposed on the polymer surface decreased. This corresponds to a reduction of the initial drug release burst observed in the case of EUD/THEOP 20/1 *w*/*w*.

Considering the obtained results, the coprecipitation was achieved only in part in the case of DICLO, for which smaller and less-defined microparticles were generally obtained with respect to the other studied drug; moreover, a burst-like effect of about 45% was observed. Well-defined spherical microparticles EUD/THEOP were instead produced, assuring a massive coprecipitation with a burst effect that reduced up to a value of 20% for EUD/THEOP 20/1 *w*/*w*. As reported in the literature [16], in this work it was also observed that the polymeric carrier has a great influence on the precipitate morphology, and by selecting the proper one, it is possible to coprecipitate even an active principle which revealed to be a bad candidate for SAS micronization, as occurred with THEOP. Moreover, different results were obtained with different drugs even using the same polymer, proving once again that the interactions between polymer, active principle, scCO_2_, and liquid solvent can influence the high-pressure vapor-liquid equilibria and modify the processability of materials.

## 4. Conclusions

In this work, it was demonstrated that Eudragit L100-55 is an effective carrier for SAS coprecipitation as composite microspheres EUD/diclofenac and EUD/theophylline were produced; the operating conditions have to be accurately selected depending on the drug used because the drug can influence the high-pressure vapor-liquid equilibria. Dissolution tests showed that the release of the drugs was significantly delayed, up to 28 and 57 times for DICLO and THEOP, respectively; therefore, Eudragit L100-55 is suitable for applications focused on controlled-release to reduce the side effects, due to drug overuse.

## Figures and Tables

**Table 1:** A list of SAS tests for Eudragit L100-55 (EUD) micronization, EUD/ diclofenac (DICLO) coprecipitation and EUD/ theophylline (THEOP) coprecipitation (C, crystals; NP, nanoparticles; MP, microparticles; cMP, coalescing microparticles; EMP, expanded microparticles); when in a single test more than one morphology is obtained, * indicates the one whose diameter is calculated.

#	Polymer/Drug [*w*/*w*]	P, [Mpa]	C_tot_ [mg/mL]	M	m.d. ± s.d. [μm]
EUD					
1	1/0	9	20	MP	1.99 ± 0.49
2	1/0	10	20	MP	1.69 ± 0.51
3	1/0	12	20	cMP	1.64 ± 0.72
4	1/0	10	40	MP	1.95 ± 0.54
EUD/DICLO					
5	0/1	9	20	NP	0.14 ± 0.05
6	20/1	9	40	MP * + cMP	* 2.16 ± 0.69
7	20/1	10	40	MP	2.47 ± 0.71
8	20/1	10	20	cMP	-
9	20/1	10	50	MP	2.92 ± 0.81
10	10/1	10	50	MP	1.53 ± 0.45
EUD/THEOP					
11	0/1	9	20	C	-
12	20/1	10	40	C	-
13	20/1	10	20	C + MP *	* 6.79 ± 1.84
14	20/1	12	20	MP	5.93 ± 1.62
15	20/1	15	20	cMP	1.64 ± 0.32
16	20/1	12	40	MP	5.65 ± 1.66
17	10/1	12	40	MP * + EMP	* 3.75 ± 1.08
